#  Psychometric properties of the Calgary Cambridge guides to assess communication skills of undergraduate medical students 

**DOI:** 10.5116/ijme.5454.c665

**Published:** 2014-12-06

**Authors:** Anne Simmenroth-Nayda, Stephanie Heinemann, Catharina Nolte, Thomas Fischer, Wolfgang Himmel

**Affiliations:** Department of General Practice, Family Medicine, University of Göttingen, Germany

**Keywords:** undergraduate medical education, questionnaires, physician-patient relations, teaching, observer variation

## Abstract

**Objectives:**

The aim of this study was to analyse the psychometric properties of the short version of the Calgary Cambridge Guides and to decide whether it can be recommended for use in the assessment of communications skills in young undergraduate medical students.

**Methods:**

Using a translated version of the Guide, 30 members from the Department of General Practice rated 5 videotaped encounters between students and simulated patients twice. Item analysis should detect possible floor and/or ceiling effects. The construct validity was investigated using exploratory factor analysis. Intra-rater reliability was measured in an interval of 3 months, inter-rater reliability was assessed by the intraclass correlation coefficient.

**Results:**

The score distribution of the items showed no ceiling or floor effects. Four of the five factors extracted from the factor analysis represented important constructs of doctor-patient communication The ratings for the first and second round of assessing the videos correlated at 0.75 (p < 0.0001). Intraclass correlation coefficients for each item ranged were moderate and ranged from 0.05 to 0.57.

**Conclusions:**

Reasonable score distributions of most items without ceiling or floor effects as well as a good test-retest reliability and construct validity recommend the C-CG as an instrument for assessing communication skills in undergraduate medical students. Some deficiencies in inter-rater reliability are a clear indication that raters need a thorough instruction before using the C-CG.

## Introduction

Acquiring communicative competence is an important goal of medical education. Especially history-taking, developing the doctor-patient-relationship, sensitive counselling, shared decision-making and breaking bad news are considered to be essential skills. Many medical faculties worldwide have integrated communication topics in a longitudinal curriculum. 1 - 5 Similar to initiatives in many other countries, the revision of the German Medical Licensure Act in 2004 emphasised the importance of teaching communicative and social skills in the medical curricula. Such skills should already be learned by younger students 6 when they begin their clinical education.

To measure whether communication skills are successfully taught, reliable instruments are needed. Several assessment instruments for communicative skills, such as the Maastricht History-taking and Advice Scoring list consisting of global items (MAAS-Global), the Liverpool Communication Skills Assessment Scale (LIV-MAAS), the Liverpool Communication Skills Assessment Scale (LCAS) and the Calgary-Cambridge Guide (C-CG), have become well-established in many countries. 7 - 10

These instruments were often developed as observation guides for the purposes of delineating evidence-based skills and enhancing detailed, descriptive, verbal feedback during the teaching and learning process. In addition, they have frequently been adapted to measure performance on summative exams such as OSCEs and are used to compare learner performance before and after a defined teaching term.

The instruments differ in form, scope and objectives. The MAAS-Global Rating List, 7 a comprehensive scale, includes 47 items with a 7-point-scale, divided into 3 sections consisting of items for assessing both communication and clinical examination skills. It was developed and validated in Dutch and in English and―after adding 27 items―converted into the LIV-MAAS Scale, 8 especially for British purposes. The Liverpool Communication Skills Assessment Scale (LCSAS) 9 is a rather short instrument with 12-items and a 4-point-scale, mainly designed for assessing OSCEs and giving student feedback during teaching. Other instruments focus on specific patient groups, such as the Structured Communication Adolescent Guide (SCAG) 14 for training communication with adolescents and their parents. These instruments did not meet our needs for assessing a younger student’s communication skill, due to their size and scope. In contrast, the Calgary Cambridge Guide (C-CG) 13 first published in 1996 in Canada, seemed to fit for our purposes.

The C-CG was developed for several reasons: first of all, it was the basis for curricular planning and defining teaching goals in communication skills. The C-CG covers the whole medical interview and was used as an observation guide during teaching. It is also used as an assessment tool, typically in short versions of the original instrument. In 2001, the C-CG became part of the Kalamazoo Consensus Statement. 15 , 16 This underlines the acceptance of the instrument within an international leading declaration for teaching communication skills. 17 Especially for a basic skill course which does not include physical examination, the 28-item version of the C-CG seems appropriate. Although the original C-CG has already been introduced and validated in several translated versions, 18 its psychometric properties have not been analysed when used in educational contexts with younger medical students.

The aim of this study was to analyse the psychometric properties of the short version of C-CG and to decide whether it can be recommended for use in the assessment of communications skills in young undergraduate medical students. Especially four aspects should be studied in detail: 

Item distribution, i. e. does the C-CG provide a differentiated assessment?

Construct validity, i. e. does the C-CG represent meaningful aspects of communication?

Test-retest reliability, i. e. can the C-CG be used reliably from semester to semester?

Inter-rater reliability, i. e. can the C-CG be used intuitively by raters?

## Methods

### Context

At Göttingen University Medical School the “basic clinical skills course” includes manual skills (e.g. injections, EKG, wound-suturing) and communication skills (such as history-taking and basic communication techniques). We use, among others, role plays and consultations with simulated patients (SP) in small-group learning sessions. The course extends over 12 weeks with 3-hour modules. Students attend this course in the beginning of their 3rd year. 

### The instrument

We chose the C-CG version with 28 items, designed for assessing the history-taking interview. 13 This version is has a 3-point scale (“no”, “yes, but”, “yes”) and is sub-divided into 6 parts: ‘initiating the session’, ‘gathering information’, ‘understanding the patient perspective’, ‘providing a structure for the consultation’, ‘building a relationship’, and ‘closing the session’.

After consulting Suzanne Kurtz, author of the C-CG, 3 researchers with a good command of English independently translated this version into German (“forward” translation). Then, a native speaker (SH) translated this preliminary instrument “backward” into English. Two senior lecturers (AS, TF), reviewed all translations and developed the pre-final version. If the versions disagreed, they consulted WH and CN. 

The final version was pre-tested with a group of student tutors in our department. The raters reported major difficulties with the 3-point scale in the original version. They had the feeling a larger selection of ratings would make assessment easier. As a consequence, a 5-point scale (based upon the typical German grading structure with 1 = excellent and 5 = deficient) was implemented.

### Preparation of the material

From a pool of 117 SP consultation videos that are routinely generated by our “basic medical skills” course, a sample of 5 videos was selected to represent the range of the quality of student performance between “excellent” and “deficient”. Two authors (AS and TF) screened the video material and selected 5 video consultations which showed a stepwise grading from excellent to deficient performances. The videotapes were converted to digitised files on DVD. 

### Participants and training 

Members from the Institute of General Practice (medical doctors, sociologists, psychologists, and student tutors) were asked to take part in the study as raters. The group was trained in a 90-minute session, including a short presentation of the experiment and the C-CG. Afterwards, an 8-minute-video, presenting a consultation between an SP and a student of the current course was shown and the group-members carried out an individual rating with the C-CG. These individual ratings were then discussed item per item with the whole group; the aim was a best possible consensus about scoring. The training was conducted by AS and TF. After this instruction, all raters received a DVD with the 5 selected SP-consultations and the C-CG in printed form. They were instructed to score the videos within the following 4 weeks. We reminded them by e-mail and telephone call. After 3 months, the rating procedure was repeated.

The ethical review board of the University of Göttingen reviewed and approved the study protocol (No. 27714An).

### Statistical analysis

All analyses were performed using SAS 9.3. Several methods were applied to assess the psychometric qualities of the C-CG:

### Item analysis

Mean scores, standard deviations (SD), ranges, and percentages of the scores given by the raters were calculated to evaluate score distributions, especially to detect possible floor and/or ceiling effects.

### Construct validity

The validity of the C-CG construct was investigated by an exploratory factor analysis. 19 The underlying factors were identified by means of varimax rotation.

### Test-retest reliability

Intra-rater reliability was measured within an interval of 3 months. The correlation between the two rating rounds was assessed with 3 different statistical measures: (1) Pearson’s r, (2) a t-test for dependent samples to analyse whether the difference between the two assessments was significantly different from zero, and (3) a descriptive analysis of how often a rater gave the same score at the 2 assessments, how often the assessments differed by 1 point and how often by 2 points or more.

### Inter-rater reliability

 Since more than 2 raters were engaged in the assessment and because more than 1 video had to be assessed, the intraclass correlation coefficient (ICC) was adequate to assess inter-rater reliability. 20 A SAS macro by Robert M. Hamer was invoked to calculate the ICC (http://support.sas.com/kb/25/031.html).

## Results

A total of 30 participants took part in the training session and each of them rated the 5 videos twice within an interval of 12 weeks. 

### Item characteristics

Table 1 shows the measures of distribution of the scores for all 28 items, the 5 scales and the overall score of the C-CG, summed for all raters and all videos for the first rating round. The means are slightly skewed to the upper end of the scale, but the raters made use of all scores and the IQR ranges as well as the 10% to 90% ranges were rather broad. The characteristics of the values for the second assessment were nearly identical (data not shown).

**Table 1.** Item characteristics

Item	Mean	SD	Min	Max	IQR ^*^	10% - 90%^**^
Greets patient	1.7	0.9	1	5	1	1 - 3
Introduces self and role	2.4	1.3	1	5	2	1 - 5
Demonstrates respect	1.8	1.0	1	5	1	1 - 3
Identifies and confirms problems list	2.4	1.2	1	5	2	1 - 4
Negotiates agenda	3.9	1.2	1	5	2	2 - 5
Scale “beginning the session”	2.4	0.7	1.0	4.2	1.0	1.5 - 2.5
Encourages patient to tell story	2.0	1.2	1	5	2	1 - 4
Appropriately moves from open to closed questions	2.4	1.2	1	5	2	1 - 4
Listens attentively	1.8	1.0	1	5	1	1 - 3
Facilitates patient’s responses verbally and non-verbally	2.1	1.0	1	5	2	1 - 4
Uses easily understood questions and comments	1.7	0.9	1	5	1	1 - 3
Clarifies patient’s statements	2.4	1.1	1	5	2	1 - 4
Establishes dates	2.0	1.0	1	5	2	1 - 3
Scale “gathering information”	2.1	0.9	1.0	4.5	1.1	1.0 - 3.6
Determines and acknowledges patient’s ideas re cause	2.7	1.4	1	5	3	1 - 5
Explores patient’s concerns re problem	2.4	1.2	1	5	2	1 - 4
Encourages expression of emotions	2.5	1.2	1	5	1	1 - 4
Picks up/responds to verbal and non-verbal clues	2.6	1.0	1	5	1	1 - 4
Scale “understanding patient’s perspective”	2.6	2.6	1.0	5.0	1.5	1.3 - 4.3
Summarises at end of a specific line of inquiry	3.1	1.2	1	5	2	1 - 5
Progresses using transitional statements	2.6	1.1	1	5	1	1 - 4
Structures logical sequence	2.4	1.0	1	5	1	1 - 4
Attends to timing	2.1	1.0	1	5	2	1 - 4
Scale “providing structure to consultation”	2.6	0.9	1.0	5.0	1.2	1.5 - 3.8
Demonstrates appropriate non-verbal behaviour	2.0	1.0	1	5	2	1 - 3
If reads or writes, doesn’t interfere with dialogue/rapport	2.1	1.1	1	5	2	1 - 4
Is not judgemental	2.0	1.1	1	5	2	1 - 4
Empathises with and supports patient	2.3	1.2	1	5	2	1 - 4
Appears confident	2.1	1.0	1	5	2	1 - 3
Scale “building relationship”	2.1	0.9	1.0	4.2	1.4	1.0 - 3.4
Encourages patient to discuss any additional points	2.5	1.4	1	5	3	1 - 5
Closes interview by summarising briefly	3.0	1.3	1	5	2	1 - 5
Contracts with patient re next steps	1.9	1.0	1	5	1	1 - 4
Scale “closing the session”	2.5	1.0	1.0	5.0	1.7	1.4 - 3.7
Overall score	2.4	0.7	-	-	1.1	1.5 - 3.5

^*^Interquartile range (difference between upper and lower quartile), ^**^10% to 90% interval

### Construct validity

On the basis of the ‘eigenvalue’ criterion (>1.0), we were able to extract 5 factors. This solution is shown in Table 2 with the corresponding factor scorings after varimax rotation. One factor comprised only 1 item (‘negotiates agenda’). The four other factors seem to represent important constructs of doctor-patient communication: technicalities of opening and closing a session with a patient, structuring the consultation, formal aspects of communication and patient orientation. However, the number of items of each factor is far from being optimal. While there were many items loading on factor 1, especially most or all items of the scale ‘gathering information’ and the scale ‘understanding patient’s perspective’, only three or fewer items loaded on factor 3 and 4. The 5-factor solution explained 74.1% of the whole variance (factor 1: 30.9%; factor 2: 15.75%; factor 3: 14.6%; factor 4: 7.4%; factor 5: 5.5%).

**Table 2:** Factor loadings for the final 5-factor solution

Items	Component				
	1	2	3	4	5
Greets patient	0.11	0.25	0.08	**0.83**	-0.11
Introduces self and role	0.06	-0.08	0.03	**0.86**	0.25
Demonstrates respect	**0.72**	0.18	0.43	0.25	-0.15
Identifies and confirms problems list	**0.47**	0.35	0.37	0.19	0.07
Negotiates agenda	-0.10	0.23	0.05	0.09	**0.81**
Encourages patient to tell story	**0.64**	0.34	0.29	0.30	-0.15
Appropriately moves from open to closed questions	**0.64**	0.36	0.43	0.19	-0.07
Listens attentively	**0.66**	0.24	0.44	0.12	-0.26
Facilitates patient’s responses verbally and non-verbally	**0.64**	0.27	0.45	0.20	-0.15
Uses easily understood questions and comments	**0.65**	0.04	0.50	0.10	-0.11
Clarifies patient’s statements	**0.74**	0.21	0.27	-0.01	0.27
Establishes dates	0.35	0.03	**0.74**	0.14	0.15
Determines and acknowledges patient’s ideas re cause	**0.79**	-0.12	0.07	-0.17	0.35
Explores patient’s concerns re problem	**0.83**	0.37	0.03	0.09	0.00
Encourages expression of emotions	**0.86**	0.21	0.13	0.08	-0.03
Picks up/responds to verbal and non-verbal clues	**0.78**	0.30	0.25	0.05	0.11
Summarises at end of a specific line of inquiry	0.31	**0.65**	0.24	0.07	0.41
Progresses using transitional statements	0.43	**0.61**	0.46	0.17	0.06
Structures logical sequence	0.29	0.38	**0.75**	-0.02	0.14
Attends to timing	0.14	0.30	0.71	-0.04	0.01
Demonstrates appropriate non-verbal behaviour	**0.63**	0.41	0.44	0.11	-0.13
If reads or writes, doesn’t interfere with dialogue/rapport	0.42	**0.58**	0.38	-0.16	-0.21
Is not judgemental	**0.67**	0.28	0.42	-0.03	-0.17
Empathises with and supports patient	**0.78**	0.41	0.19	0.11	-0.07
Appears confident	0.15	**0.59**	0.42	0.07	0.07
Encourages patient to discuss any additional points	0.45	**0.56**	0.01	0.40	-0.10
Closes interview by summarising briefly	0.10	**0.72**	0.16	0.15	0.35
Contracts with patient re next steps	0.46	**0.68**	0.18	0.00	0.04

Bold values indicates variables with significant scorings of at least 0.47.

### Test-retest reliability

The raters’ mean total score at the first assessment was 2.37 (SD 0.7, median 2.3, range 1.2 to 4.2). The raters gave somewhat lower, i. e. better, scores at the second assessment (mean 2.26, SD 0.7, median 2.2, range 1.1 to 4.1). The t-test for the difference between the 2 assessments, although marginal (0.11, 95%CI [0.01, 0.17]), was statistically significant (p = 0.023).

The ratings at the first and second rating round correlated at 0.75 (Pearson’s r, p<0.0001). In 34.1 to 66.7% in-stances, the scores of both assessments of a rater were identical (data not shown). The items with the best agreements between a rater’s first and second rating were: ‘closes interview by summarising briefly’ (agreement in 66.7 % of instances) and greets patient (62.2%); items with the worst agreement were: ‘Identifies and confirms problems list’ (34. 1%); ‘if reads or writes, doesn’t interfere with dialogue/rapport’ (in 36.3%) and ‘determines and acknowledges patient’s ideas re cause’ (37%). Correspondingly, differences of more than 1 point could be observed quite frequently for the following items: ‘if reads or writes, doesn’t interfere with dialogue/rapport’ (31.9%); ‘identifies and confirms problem list’ (28.2%); ‘determines and acknowledges patient’s ideas about cause’ (28.2%); ‘summarises at end of a specific line of inquiry’ (24.5%).

**Table 3.** Intraclass Correlation (ICC)

Items	First assessment			Second assessment	
	ICC	95%CI		ICC	95%CI
Greets patient	0.21	(0.07; 0.71)		0.31	(0.12; 0.80)
Introduces self and role	0.25	(0.09; 0.75)		0.28	(0.11; 0.77)
Demonstrates respect	0.43	(0.21; 0.87)		0.44	(0.21; 0.87)
Identifies and confirms problems list	0.27	(0.10; 0.76)		0.19	(0.06; 0.68)
Negotiates agenda	0.10	(0.03; 0.51)		0.15	(0.05; 0.61)
Encourages patient to tell story	0.57	(0.31; 0.92)		0.37	(0.17; 0.84)
Appropriately moves from open to closed questions	0.42	(0.20; 0.86)		0.30	(0.12; 0.79)
Listens attentively	0.52	(0.27; 0.90)		0.38	(0.17; 0.84)
Facilitates patient’s responses verbally and non-verbally	0.38	(0.16; 0.84)		0.27	(0.11; 0.77)
Uses easily understood questions and comments	0.25	(0.09; 0.74)		0.17	(0.05; 0.65)
Clarifies patient’s statements	0.16	(0.05; 0.63)		0.05	(0.00; 0.36)
Establishes dates	0.17	(0.05; 0.65)		0.08	(0.02; 0.48)
Determines and acknowledges patient’s ideas re cause	0.18	(0.06; 0.67)		0.15	(0.04; 0.62)
Explores patient’s concerns re problem	0.27	(0.10; 0.76)		0.19	(0.07; 0.68)
Encourages expression of emotions	0.26	(0.10; 0.75)		0.20	(0.07; 0.68)
Picks up/responds to verbal and non-verbal clues	0.26	(0.10; 0.75)		0.25	(0.09; 0.75)
Summarises at end of a specific line of inquiry	0.25	(0.09; 0.75)		0.19	(0.07; 0.68)
Progresses using transitional statements	0.37	(0.16; 0.83)		0.28	(0.11; 0.78)
Structures logical sequence	0.34	(0.14; 0.82)		0.24	(0.09; 0.74)
Attends to timing	0.40	(0.18; 0.85)		0.16	(0.05; 0.63)
Demonstrates appropriate non-verbal behaviour	0.49	(0.25; 0.89)		0.33	(0.14; 0.81)
If reads or writes, doesn’t interfere with dialogue/rapport	0.44	(0.21; 0.87)		0.30	(0.12; 0.79)
Is not judgemental	0.45	(0.22; 0.88)		0.27	(0.10; 0.77)
Empathises with and supports patient	0.41	(0.19; 0.86)		0.37	(0.16; 0.83)
Appears confident	0.28	(0.11; 0.78)		0.17	(0.05; 0.63)
Encourages patient to discuss any additional points	0.44	(0.21; 0.87)		0.28	(0.11; 0.77)
Closes interview by summarising briefly	0.17	(0.05; 0.65)		0.28	(0.11; 0.77)
Contracts with patient re next steps	0.30	(0.12; 0.79)		0.35	(0.15; 0.82)

### Inter-rater reliability

The ICCs for each item were only moderate and ranged from 0.05 to 0.57 (Table 3) . Items with an ICC above 0.4 at the first rating round were: ‘encourages patient to tell story’, listens attentive’ demonstrates respect’, ‘demonstrates appropriate non-verbal behaviour’ and ‘is not judgmental’. The items ‘demonstrates respect’ and ‘listens attentively’ scored quite highly at the second rating round. The ICC for the following items scored worse than 0.2 either at the first or the second or at both rating rounds: ‘negotiates agenda’, ‘clarifies patient’s statements’, ‘determines and acknowledges patient’s ideas’.

## Discussion

Reasonable score distributions of most items without ceiling or floor effects as well as a good test-retest reliability and construct validity recommend the C-CG as an instrument for assessing communication skills in undergraduate medical students and for regularly monitoring the success of the communication skills curriculum. Some deficiencies in inter-rater reliability are a clear indication that raters need a thorough instruction before using the C-CG. 

### Comparison with literature and meaning of the results

Using the C-CG, the raters exploited the range of scores for nearly all items (Table 1) . The wide range between 1 and 5 shows the ability of the C-CG to detect differences and graduations within single communicative skills. We conclude the C-CG is well-suited to identify good compared to poor performers. Several items were assessed with high reliability. For example, the item ‘demonstrates respect’ showed only a 10% disagreement by more than 1 point between first and second assessment. The ICCs for this item were higher than 0.40 and it clearly differentiated between the 5 videos and had a high scoring on the first factor. Similar effects were also observed for the items such as ‘greets patient’, ‘introduces self and role’, ‘empathises with and supports patient’ and ‘closes interview by summarising briefly’. On the basis of this psychometric analysis, we found that the C-CG is able to assess and reproduce the main learning goals in this early stage of medical education: to build a relationship and to keep in touch with a new patient by means of empathic listening and sensitive questioning.

Other items, however, reduced the psychometric qualities of inter-rater and intra-rater reliability as well as construct validity. A quite exceptional item is ‘negotiates agenda’. In 27% of instances, the raters differed from the first to the second rating round by more than 1 point. This item also has a fairly low ICC value (Table 2) , which means that raters scored the same performance quite differently. In addition, this item was the only one that scored high on the fifth factor (Table 3) . Scheffer et al. described the same problem with this item in their validation of a global rating instrument that they compared with the C-CG. When we simulate a first consultation, we assume that this is a new patient whose reason for coming to see the ‘GP’ is not yet known. Therefore, it must have been difficult for the raters to assess this item in particular. Interestingly, neither the MAAS-Global, LIV-Maas, LCSAS nor the SEGUE-Framework contain a comparable item. 7 - 9 , 21

Similar problems, although less distinct, were observed with the item ‘identifies and confirms problem list’ that showed a poor intra-rater reliability, a poor ICC and a rather low scoring on the first factor. Obviously, the raters had also difficulties with the item ‘If reads or writes, doesn’t interfere with dialogue/rapport’. In more than 30% of instances, they differed by more than 1 point between first and second assessment. These items measure skills that not so important for younger students who are just beginning to learn to build relations with new patients. History-taking is a first step in this learning process. We often encourage undergraduate students to avoid writing and reading in order to fully concentrate on the patient’s verbal and nonverbal signs. These items, therefore, do not play a major role in undergraduate medical education, at least in Germany, and seemed difficult for raters to assess.

Kurtz and Silverman 13 suggested dividing the C-CG into different sections such as ‘beginning’, ‘gathering information’, or ‘closing the session’. This structure follows the typical course of a doctor-patient encounter. This is very helpful while observing the different stages of the encounter. In contrast or additionally, our factor analysis accentuates the different aspects of communication which re-occur throughout the different chronological stages of the encounter. Our first factor comprises those aspects that characterise the typical aspects of patient-oriented behaviour such as patient encouragement, exploring patient’s concerns or demonstrating non-verbal behaviour. The second factor reflects the ability to structure the communication. The third factor focuses on formal aspects of the communication, including dates and timing. The fourth factor considers the technicalities of beginning a session with a patient. In our opinion, this structure is truly valuable because it reflects the different aspects of communication behaviour. We assume that the factors identified in our factor analysis may also be valid for other language versions of the C-CG. With regards to the one-item factor ‘negotiates agenda’, there may be cultural differences between communication expectations or the way history-taking is integrated into the healthcare system which may alter the validity of this item in other cultural settings. 

### Strengths and limitations

The raters came from a wide variety of backgrounds (student tutors, medical doctors, sociologists, psychologists), which reflects the interdisciplinary teaching staff in our medical school. The group or raters was balanced in terms of gender. This mix of raters helped to assess how the C-CG performs in real life. Although we analysed the C-CG using 300 rating assessments (5 videos x 30 raters x 2 time points), the sample size of five videos was small. For a valid inter-rater assessment, it would have been better to ask raters to evaluate a large number of student consultations which differed only marginally. However, such a procedure would have exceeded our resources. Inter-rater reliability, though not optimal according to our results, may have even been overestimated due to the small number of different consultation videos.

### Implications for practice

The C-CG seems to be an adequate instrument to assess skills and abilities that medical younger students should learn in communication courses and to assess whether teachers have successfully taught these skills. But three caveats are required:

1.Some items may limit the validity of the instrument if is intended to assess younger students or the quality of communication courses for these students. The item ‘negotiating agenda’ proved to be such a case. 

We suggest deleting this item when the C-CG is used in early stages of the medical curriculum where younger students are beginning to learn their professional role in communicating with patients. In later phases of the curriculum, it may be important to include this item since it measures typical tasks in doctor-patient encounters, which include not only talking with the patient, but also a structured procedure in diagnostics, treatment, referral and other clinical activities. 

2.Although teachers usually prefer to sum up similar items and, thus, to calculate a sum score, it could be misleading to sum up the items of the six scales of the original version because the items in each scale comprise different skills and abilities and do not represent consistent and coherent concepts, as could be shown in the factor analysis. If teachers and raters are interested to learn whether students have a good command of certain communication skills, e.g. patient-orientation, and to find out a student’s strengths and weaknesses for later interventions, they should rather look at the items of the factors that we extracted.

3.Although we trained raters to use the C-CG adequately and although most of the items of the C-CG seemed to be self-explanatory, raters had problems with several items. A more thorough training may be appropriate, especially for those items that are more difficult to assess. Since the ICCs for the second assessment were almost constantly poorer than for the first assessment, it may also be necessary the repeat the training, or at least to provide a refreshment. If the C-CG will be later used as basis for official grading, a better inter-rater reliability is important, not least to ensure fairness towards the students of an entire semester and to avoid that an individual’s assessment is dependent on a rater’s personal interpretation of the C-CG items.

### Future research

Future research should focus on the construction of the C-CG and try to re-assess and refine the underlying factor structure of this instrument. It may be necessary to create a version of the C-CG which is focused on a subset of items especially relevant for this earlier study phase. Although it is difficult to find or establish a sort of gold standard, the C-CG should be validated against such a standard in the future to determine construct validity, especially convergent validity. 

## Conclusions

Originally created for use in curricular planning and to define teaching goals for communication skills, the C-CG short version can also be recommended for evaluation purposes. A student’s communications skills can be reliably assessed with the C-CG. In addition, teachers can be regularly informed whether they have reached their training goals and whether they have become better or worse, compared to the previous semester. However, it is of upmost importance that raters be well-trained in the use of the instrument for results to be reliable. Our factor analysis indicated four separate latent concepts: patient-orientation, communication structure, formal aspects and technicalities of beginning a session with a patient. These concepts represent important features of the medical encounter and are relevant even for undergraduate students, just beginning to learn the basic communication skills involved in doctor-patient consultations.
